# Supplementary figures and images for: In-silico approaches for identification of compounds inhibiting SARS-CoV-2 3CL protease
Source: PLoS One. 2023 Apr 14;18(4):e0284301. doi: 10.1371/journal.pone.0284301 (PMC10104361; doi:10.1371/journal.pone.0284301)

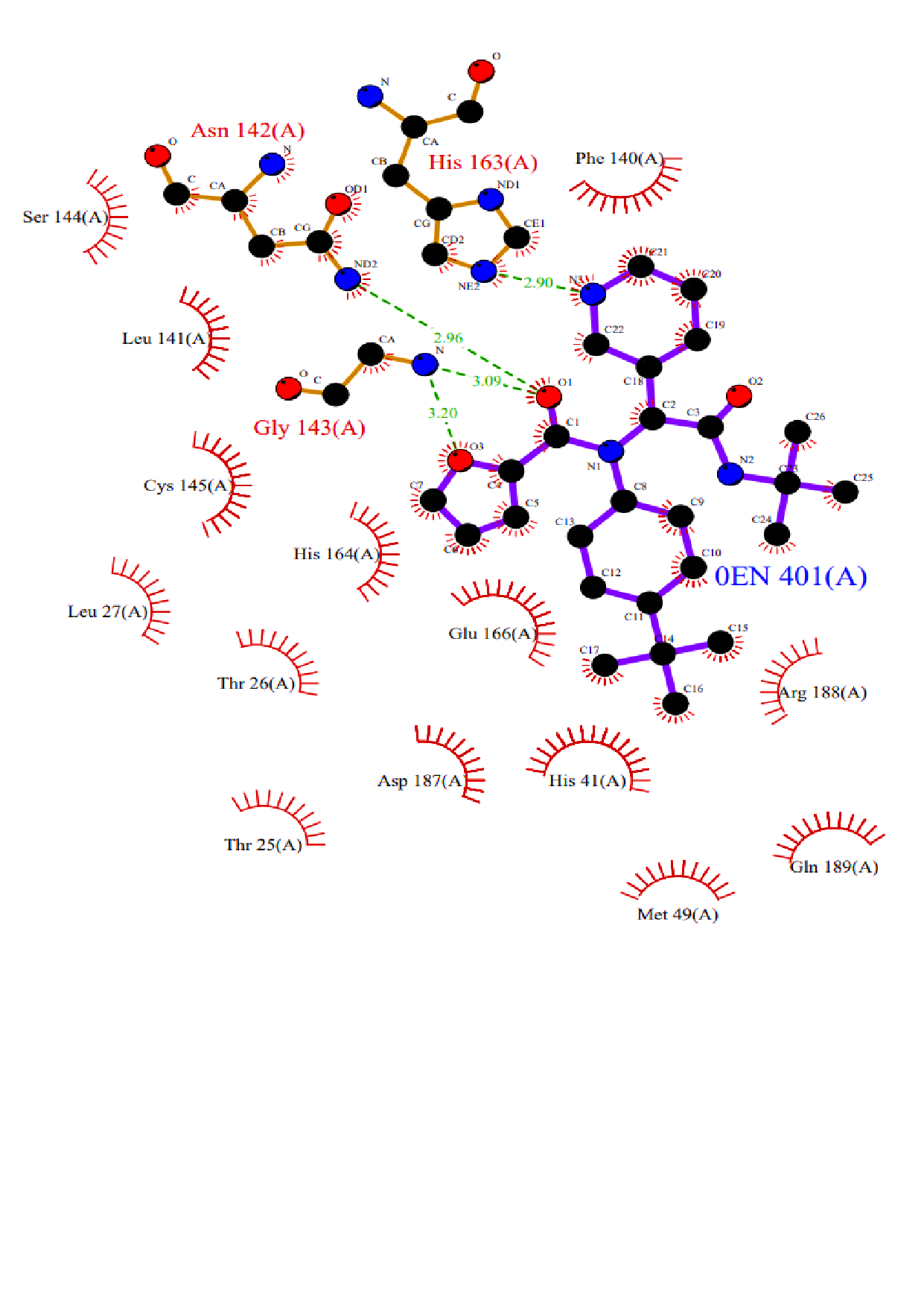

Supplement: S1 Fig — Hydrogen bonds are shown in the green dashed line. Other residues formed hydrophobic contacts. (TIF) [file pone.0284301.s001.tif]

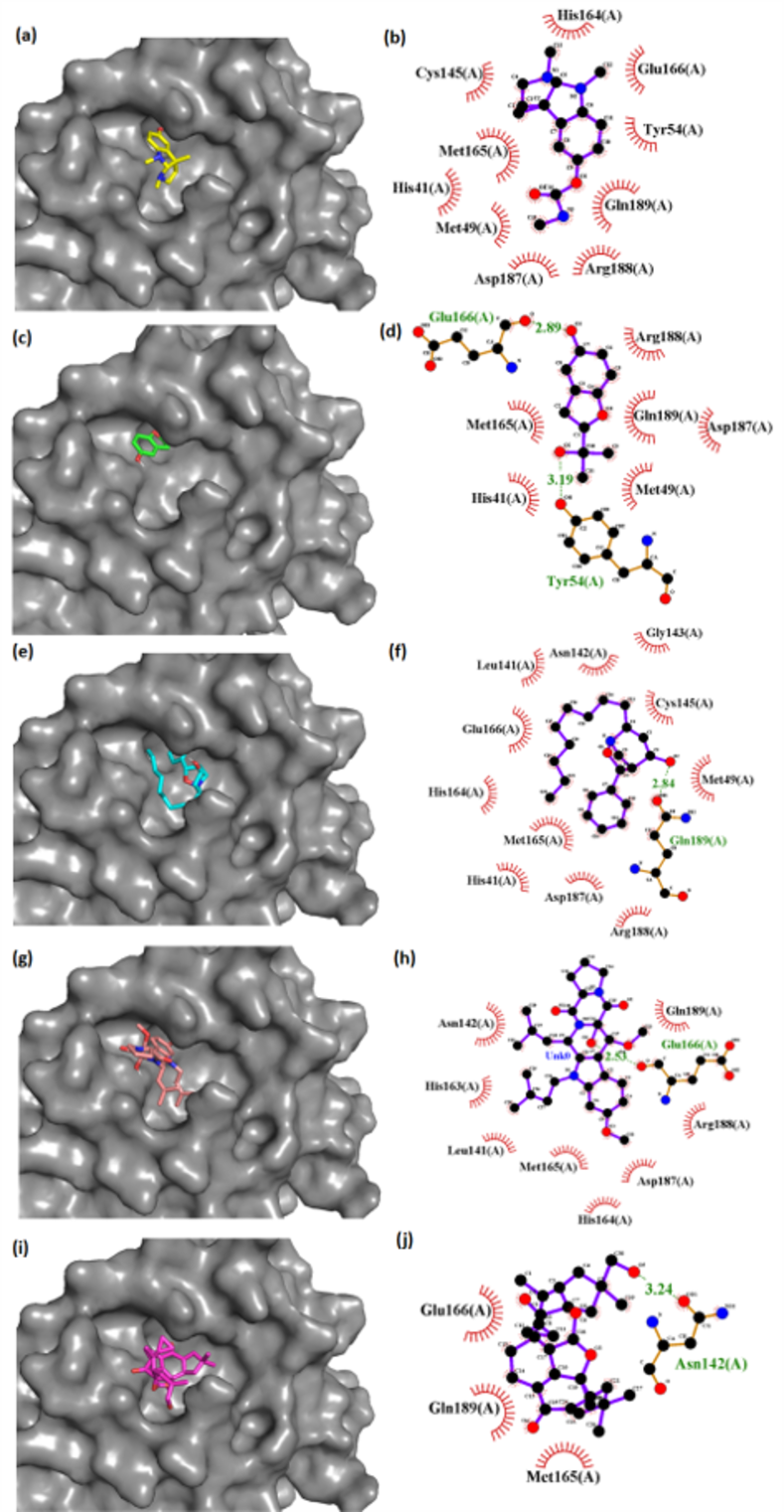

Supplement: S2 Fig — Plots are shown for the best pose generated after the molecular docking.(a, b) CMP4(NET5) (c, d) CMP10(NET5) (e, f) CMP2(NET5) (g, h) CMP9(NET1) (i, j) CMP4(NET1). (TIF) [file pone.0284301.s002.tif]

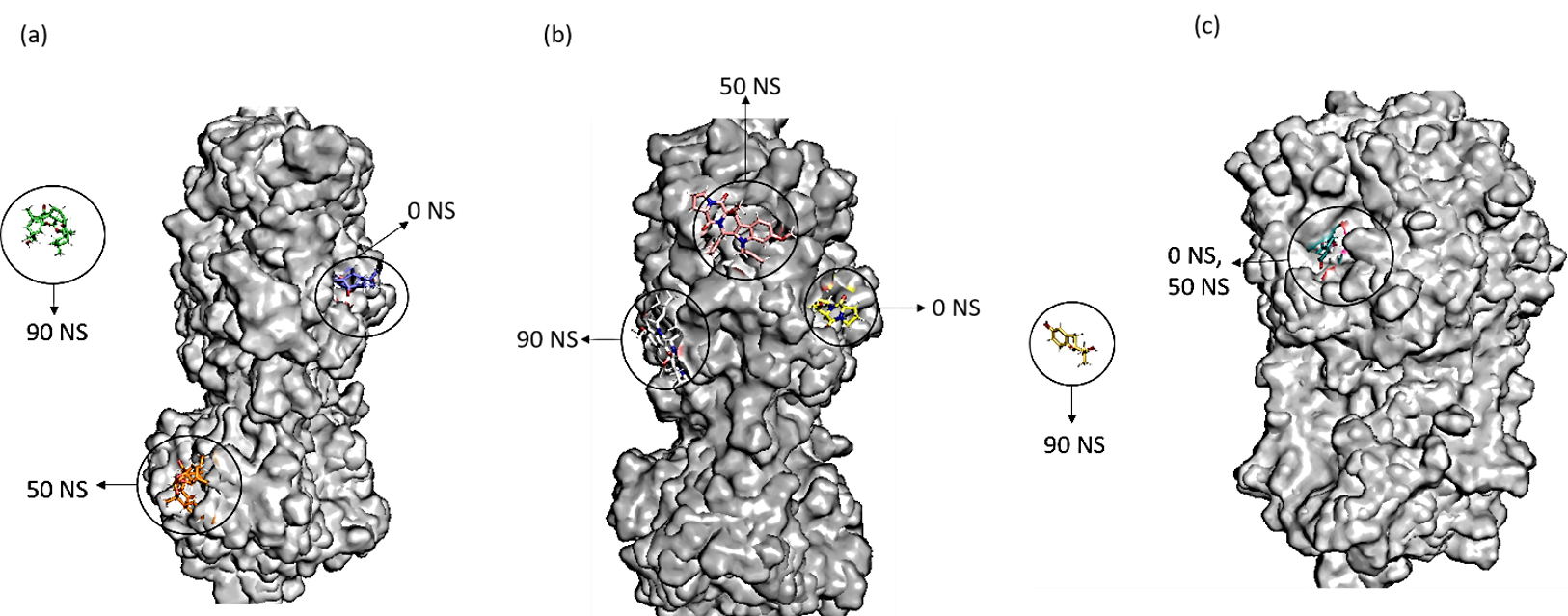

Supplement: S3 Fig — (TIF) [file pone.0284301.s003.tif]

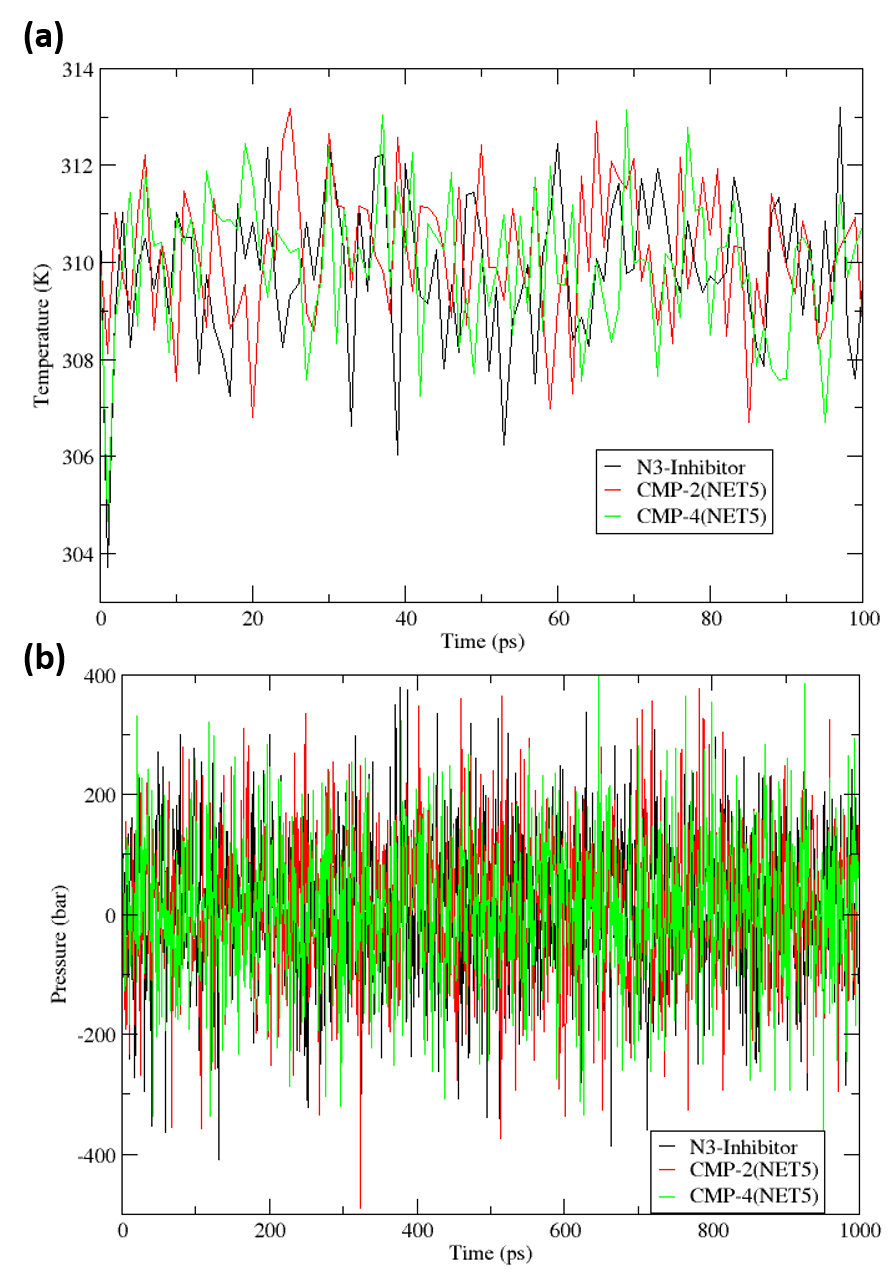

Supplement: S4 Fig — (TIF) [file pone.0284301.s004.tif]

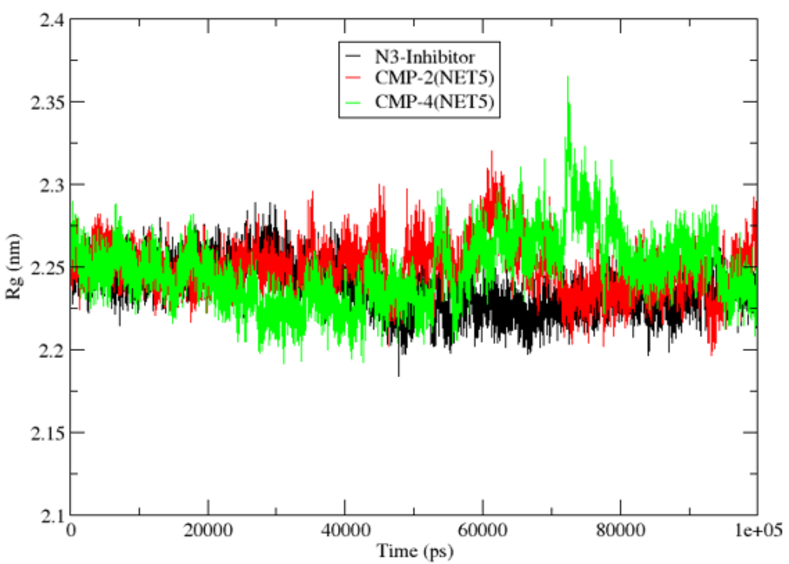

Supplement: S5 Fig — (TIF) [file pone.0284301.s005.tif]

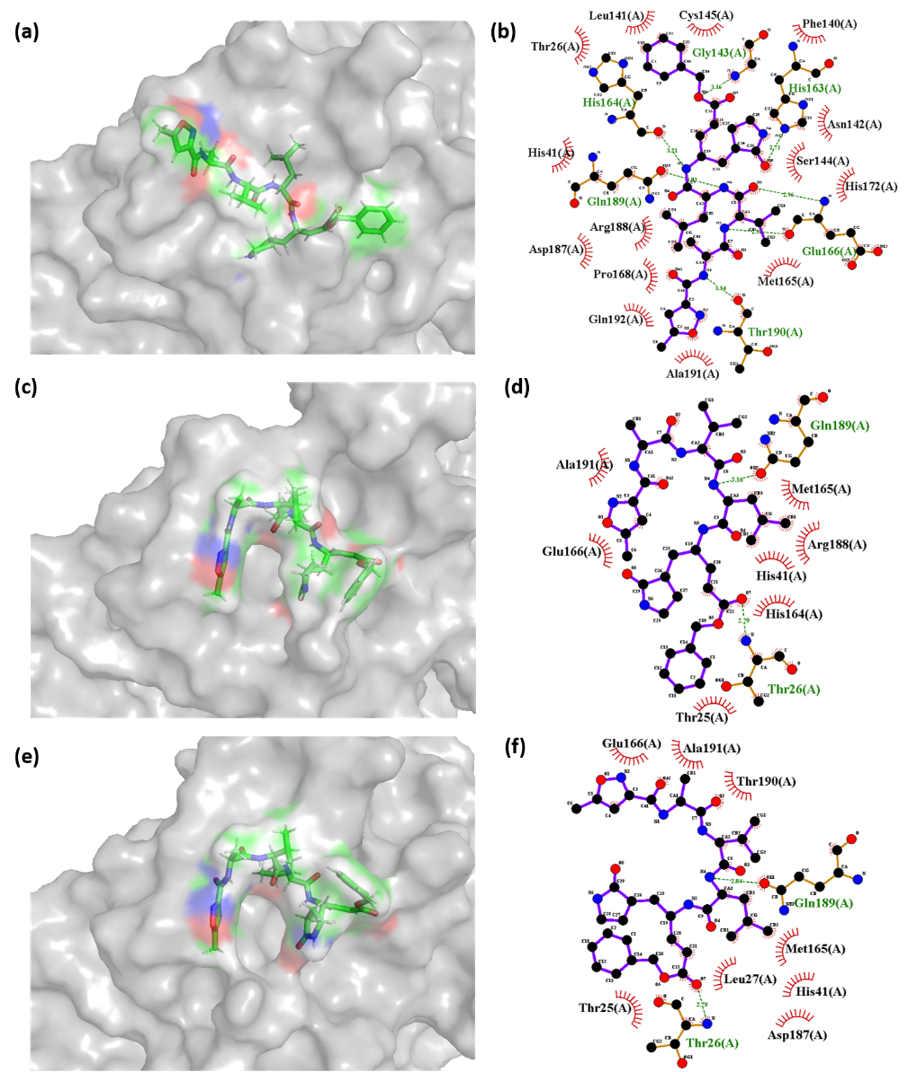

Supplement: S6 Fig — 3D and 2D interaction plot of native inhibitor N3 with the 3CL-protease protein at (a, b) 0 ns (c, d) 50 ns, and (e, f) 90 ns of the simulation trajectory. 2D interaction map was formed using LigPlus. (TIF) [file pone.0284301.s006.tif]
