# Supplementary material for: In-silico approaches for identification of compounds inhibiting SARS-CoV-2 3CL protease
Source: PLoS One. 2023 Apr 14;18(4):e0284301. doi: 10.1371/journal.pone.0284301 (PMC10104361; doi:10.1371/journal.pone.0284301)
Supplement: Attachment — Submitted filename: My response.docx [file pone.0284301.s007.docx]

Manuscript title: *In-silico* Approaches for Identification of Compounds Inhibiting SARSCoV-2 3CL Protease (Manuscript ID: PONE-D-23-03061).

This manuscript focused on finding an *in-silico* cure for COVID-19 using several tools, such as machine learning techniques with virtual drug screening methods. This manuscript is likely to meet the basic requirements of the journal. However, the manuscript is not mature, and the following points should be addressed.

1. The researchers should number the pages of the manuscript for ease of review.
2. The way you write the word “figure” in the text should be unified (Figure or figure) and bold.
3. The way you write the “IC50 and pIC50” in the text should be corrected (IC_50_ and pIC_50_).
4. The way you write the word “SARS-COV2” in the text should be unified (SARS-COV2 or SARS-COV-2).
5. The way you write the word “CMP2^(NET5)^” in the text should be unified (CMP2^(NET5)^or CMP-2^(NET5)^) and the remaining CMPs.
6. Why do the authors express the simulation duration in figures in Pico and not in Nano, as mentioned in the text?
7. The way you write the word “3CL protease” in the text should be unified (3CL protease or 3CL Protease).
8. The numbers of tables in the first paragraph below "**3.4 Hit Compounds Docking**" should be corrected as **Table 4** and **Table 5**, not **Table 3** and **Table 4**.
9. Before the title "**3.5 Molecular Dynamics Simulation Analysis**" the corrected amino acid was Gln^189,^ not Glu^189^.
10. Before title "**3.5 Molecular Dynamics Simulation Analysis**", His^41^ was present in CMP2^(NET5)^, CMP4^(NET5)^ and CMP10^(NET5)^ not only in CMP2^(NET5)^.
11. The distance before the citation number was sometimes deleted and sometimes not. Uniform.
12. The way you write the word “antiviral” in the text should be unified (antiviral or anti-viral).
13. The authors should mention the SASA, RMSF, histograms indicating the fractions of binding between the protein’s amino acids and their compounds and the numbers of hydrogen bonds that were seen in the complex formed by compounds during the 100-ns molecular dynamics simulation.
14. What is the meaning of S**N**MILES in the title of **Table 3**.?
15. The caption of **Figures 2** and **3** needs correction inside it about the codes of compounds.
16. The letter "N" at the beginning IUPAC name of the native inhibitor should be in italic.
17. Below **Figure 7**., the authors mentioned only five residues involved in H-bonding. Where is the sixth one? (His^163^).
18. Below **Figure 7**., the corrected amino acid is Gln^189,^ not Glu^189^.
19. Before **Figure 8**., in CMP4(NET5) at 50 ns, Glu166 didn’t disappeared.
20. In **Figure 8. (b)**, the amino acid Cys145 didn’t seem clear.
21. **Figure 9** didn’t mention in the text of the manuscript.
22. The table number at "Steered MD Simulation" is Six, not Five. (**Table 5** was duplicated).
23. The resolution of **Figure 9** is not suitable for publication.
24. The references style in the "reference section" must be uniform and should mention the recent references. The interval of pages should be mentioned in all references.

Finally, the MS will be suitable for publication after the above corrections/changes.
